# Sorcin regulates alveolarization and airway tissue remodeling during lung morphogenesis

**DOI:** 10.1007/s00018-025-05870-y

**Published:** 2025-10-28

**Authors:** Claudia Tito, Luciana De Angelis, Alessia Iaiza, Annalisa Pia Abbinantefina, Anna Benedetti, Gilla Mazzanti, Vincenzo Petrozza, Mattia Lauriola, Luca Tamagnone, Andrea Ilari, Silvia Masciarelli, Gianni Colotti, Francesco Fazi

**Affiliations:** 1 Department of Anatomical, Histological, Forensic & Orthopaedic Sciences, Section of Histology and Medical Embryology, Sapienza University of Rome, Rome, Italy; 2 Department of Medico-Surgical Sciences and Biotechnologies, Sapienza University of Rome, Latina, Italy; 3 Translational Oncology Research Unit, IRCCS Regina Elena National Cancer Institute, Rome, Italy; 4 Department of Experimental, Diagnostic and Specialty Medicine (DIMES), University of Bologna, Bologna, Italy; 5 Department of Life Science and Public Health, Histology and Embryology Unit, Catholic University of the Sacred Hearth, Rome, Italy; 6 Institute of Molecular Biology and Pathology, Italian National Research Council, IBPM-CNR, Rome, Italy; 7 Department of Anatomical, Histological, Forensic & Orthopaedic Sciences, Section of Histology and Medical Embryology, Sapienza University of Rome, Via A. Scarpa 14-16, Rome, 00161 Italy; 8 Institute of Molecular Biology and Pathology, Italian National Research Council, IBPM-CNR, P.le A. Moro 5, Rome, 00185 Italy

**Keywords:** Lung development, Sorcin KO, Alveolarization, Branching morphogenesis, Airway remodeling

## Abstract

Sorcin, a key calcium-sensing protein, regulates calcium concentration within the endoplasmic reticulum (ER), promoting apoptosis resistance and ER stress. It also modulates downstream signaling pathways of the epidermal growth factor receptor (EGFR), influencing cellular migration and invasion in non-small-cell lung carcinoma (NSCLC) cell lines. For this purpose, this study investigates the relationship between Sorcin and EGFR expression during lung development at the physiological level. Our study was conducted on WT and Sorcin Knock-out (*Sri*^*−/−*^) mice, where we performed various analyses, including histological examination, gene and protein expression analysis, and confocal microscopy. Our findings reveal that *Sri*^−/−^ mice, compared to wild-type controls, exhibit: (1) impaired alveolarization and abnormal development of bronchi and bronchioles, as observed in histological sections; (2) decreased expression of genes encoding branching morphogenesis markers (e.g., *Fgf10*) and surfactant proteins (e.g., *Sp-b*, *Sp-c* and *Abca3*), as shown by real-time PCR; (3) increased glycogen content decreased lipid droplets, indicative of type II pneumocyte immaturity and impaired surfactant lipid production; (4) reduced levels of EGFR, RAS and RAB5C proteins, consistent with defects in lung maturation and surfactant protein recycling, as demonstrated by Western blot analysis; and (5) increased expression of phalloidin, α-smooth muscle actin and vimentin, suggesting increased bronchial thickening associated with airway tissue remodeling. Collectively, these data reveal a novel role for Sorcin in lung alveolarization, pulmonary surfactant production, and airway remodeling associated with bronchial contractility, supporting its involvement in respiratory diseases such as respiratory distress syndrome (RDS), asthma and chronic obstructive pulmonary disease (COPD).

**Supplementary Information:**

The online version contains supplementary material available at 10.1007/s00018-025-05870-y.

## Introduction

Fetal lung development is a complex process crucial for postnatal respiratory health. Disruptions in this delicate process can lead to fetal lung development disorders, impacting neonatal outcomes and potentially influencing long-term health.

Several key factors influence fetal lung maturation, including surfactant proteins (SP-A, SP-B, SP-C, and SP-D) [1–4], the ATP-binding cassette sub-family A member 3(ABCA3) [5], growth factor (FGF-7, FGF-10, EGF and their receptors FGFR and EGFR) [3, 6–9], thyroid transcription factor-1 (TTF-1) [10], transcription factor Sox9 [11], Sonic Hedgehog [12] and ion transport (calcium and chloride, in particular) [13–16].

Lung development progresses through five highly coordinated and regulated stages: the embryonic stage (weeks 4 to 7 of gestation), the pseudoglandular stage (weeks 7–16), the canalicular stage (weeks 16–28), the saccular stage (weeks 28–36), and the alveolar stage (week 36 of gestation to early childhood) [17–19]. Between the pseudoglandular and saccular stages, the number of peripheral lung tubules increases dramatically, followed by sacculation, forming the gas exchange region of the lung. This process involves the development of an extensive pulmonary microvascular capillary bed in close proximity to the epithelial cells. Epithelial cells lining the peripheral lung saccules differentiate into large, squamous alveolar type I (ATI) cells, which comprise the majority of the alveolar surface and facilitate gas exchange. Smaller, cuboidal alveolar type II (ATII) cells, which constitute approximately two-thirds of the alveolar epithelium, produce pulmonary surfactant. Pulmonary surfactant, a mixture of lipids (primarily phospholipids like phosphatidylcholine and phosphatidylglycerol) and proteins (SP-B, SP-C, SP-A and SP-D), is essential for reducing surface tension at the air-liquid interface following the onset of ventilation at birth, enabling efficient gas exchange, maintaining alveolar stability, and preventing end-expiratory atelectasis (lung or lobe collapse). Pulmonary surfactant deficiency due to immaturity is the primary cause of respiratory distress syndrome (RDS) in premature infants, leading to breathing difficulties and poor oxygenation shortly after birth [9, 18]. Surfactant production and release by ATII cells is a complex process. Phospholipids and surfactant proteins are packaged into cellular structures known as lamellar bodies (LBs), a process involving early endosomes and the formation of small vesicles. Fusion of LBs with the ATII plasma membrane, a Ca^2+^-dependent mechanism, triggers the exocytosis of pulmonary surfactant into the alveolar airspace. Secreted surfactant can be recycled (25–95%) by ATII cells and re-secreted into the alveolar lumen [20] or degraded by alveolar macrophages [21, 22]. ABCA3, an ATP-binding cassette (ABC) transporter highly expressed in ATII cells, is crucial for the transport of lipid surfactant into LBs. *Abca3* inactivation in mice results in respiratory failure, surfactant loss, depletion of lung phosphatidylglycerol and impaired LB formation [23, 24]. Mutations in the *Abca3*,* Sp-b*,* and Sp-c* genes are associated with congenital respiratory disorders in infants, children, and adults, leading to respiratory failure. Histological features of affected lung tissue of these patients include reduced or absent LBs, accumulation of eosinophilic material and alveolar macrophages, increased periodic acid-Schiff (PAS) staining (indicating pneumocyte immaturity), thickened alveolar septa, and increased cellularity [4, 25, 26]. EGFR is essential for lung development. *Egfr*^−/−^ mice (as those of the *Abca3*^−/−^ mice) exhibit an RDS-like phenotype, with immature lungs, collapsed alveoli, thickened alveolar septa, and insufficient surfactant production. EGFR inactivation also leads to ATII cell immaturity, characterized by increased glycogen content and reduced LB numbers. These alveolarization defects result from impaired branching morphogenesis; *Egfr*^*−/−*^ mice show dilated bronchi with fewer tubules and increased mesenchyme [3, 8, 9, 19]. Sorcin is a highly expressed calcium-binding protein, ranking among the top 10% of proteins by expression in the lung (source: PaxDb 5.0). Ca^2+^ signaling triggers LB exocytosis and surfactant secretion [21]. Increased cytoplasmic calcium concentration ([Ca^2+^]_c_), due to Ca^2+^ release from intracellular stores and Ca^2+^ influx from extracellular space, drives LB fusion with the plasma membrane, triggering their exocytosis and surfactant secretion [27–30]. Sorcin regulates calcium homeostasis by modulating several calcium channels/pumps/exchangers, including the sarco/endoplasmic reticulum Ca^2+^ pump (SERCA), the sarcolemmal Na^+^-Ca^2+^ exchanger (NCX), the plasma membrane Ca^2+^ pump (PMCA), the ryanodine receptors (RyRs) and the L-type voltage-gated calcium channel (LVCC), which are involved in branching morphogenesis and surfactant production. Sorcin also interacts with annexin-7 (a Ca^2+^-dependent membrane binding protein important in membrane fusion during exocytosis), protein kinase A and Ca^2+^/calmodulin-dependent kinase, all of which are involved in surfactant secretion [27, 30–41]. Furthermore, Sorcin regulates the expression of ATP-dependent ABC efflux pumps like ABCB1 and ABCB4, influencing small molecule export and multidrug resistance in cancers [32, 42].

We already demonstrated that Sorcin and EGFR expression are significantly correlated and associated with reduced overall survival in cancer patients. Mechanistically, Sorcin directly binds EGFR protein in a calcium- dependent fashion and regulates calcium (dys)homeostasis linked to EGF-dependent EGFR signaling. Moreover, Sorcin controls EGFR proteostasis and signaling and increases its phosphorylation, leading to increased EGF-dependent migration and invasion. Silencing of Sorcin cooperates with EGFR inhibitors in the regulation of migration, highlighting calcium signaling pathway as an exploitable target to enhance the effectiveness of EGFR-targeting therapies [43]. Of note, Sorcin knockout (*Sri*^*−/−*^) reduces EGFR levels in the bronchiolar region of mice lungs [43].

These results prompted us to analyze the relationship between EGFR and Sorcin at the physiological levels, investigating lung development and surfactants homeostasis during lung morphogenesis using *Sri*^−*/−*^ mouse model compared to wild-type controls. Finally, since intracellular calcium regulates airway smooth muscle contraction, we explored Sorcin’s role in airway tissue remodeling associated with altered respiratory function.

## Materials and methods

### Mouse models

*Sri*^−*/−*^ mice were provided by Hèctor H. Valdivia and Carmen R. Valdivia (Department of Internal Medicine, Division of Cardiovascular Medicine, University of Michigan, Ann Arbor, MI 48109, USA). These mice were generated as described in the work of Chen et al. [44].

C57BL/6 wild-type mice were purchased from Jackson laboratory (Bar Harbor, ME, USA) and were housed in the Histology Department-accredited animal facility. All the procedures were approved by the Italian Ministry for Health and were conducted according to the US National Institutes of Health (NIH) guidelines (Approval number: 605/2023-PR).

### Tissue mouse collection and hematoxylin/eosin staining

Lung tissues were harvested from E13.5 and E16.5 embryos, 3-week-old, 6-week-old, and 3-month-old mice following cervical dislocation. For histological analysis, tissues were fixed in 4% paraformaldehyde overnight, dehydrated in ethanol, cleared in xylene at room temperature and then, embedded in paraffin. Formalin-fixed paraffin-embedded (FFPE) lung tissue samples were sectioned at 10 μm thickness into SuperFrosts Plus-slides using a Leica RM2255 microtome. Sections were deparaffinized in xylene and rehydrated gradually up to 100% ethanol. Morphological analyses were performed using routine hematoxylin and eosin staining or periodic acid Schiff (Sigma-Aldrich, #395B) staining. The ratio of air-filled space on total lung area (3-weeks old, *n* = 5 mice per condition) and the bronchiolar thickness average (6-week-old and 3-month-old, *n* = 4 mice per condition) were quantified by ImageJ software.

### Oil red and nile red assay

Cryosection OCT-frozen lung tissues (3-weeks old) were cut into SuperFrosts Plus-slides by using a Leica cryostat. They were fixed in formalin buffered solution 10% (Sigma-Aldrich) for 10 min at room temperature (RT), then washed in tap water and stained with oil red solution (Sigma-Aldrich, #O-0625) or nile red solution 485/540 at the final concentration of 1ug/ml (#19123-Sigma-Aldrich). (Images were acquired under the microscope for analysis and quantification of lipid droplets was performed by ImageJ software. *n* = 4 mice per condition were analysed.

### Total RNA extraction from tissues, cDNA reverse transcriptase and RT-qPCR

Total lung OCT-frozen tissues (3-week-old) were homogenized in 500 ml of TRIzol RNA Isolation System (Invitrogen) and RNA was extracted using the according to manufacturer instructions. Reverse transcription to cDNA was performed with the High-Capacity RNA-to-cDNA Kit (Applied Biosystems), and cDNA was amplified using the SYBR TM Green PCR Master Mix (Thermo Fisher Scientific) on QuantStudioTM 7 Flex Real-Time PCR System, 384 well (Applied Biosystems). The relative expression values were normalized using housekeeping H3 gene. The following oligo sequences were used: *H3* FW: 5 ´-GTGAAGAAACCTCATCGTTACAGGCCTGGT-3 ´; *H3* RW: 5’- CTGCAAAGCACCAATAGCTGCACTCTGGAA-3’; *Fgf10* FW: 5’-GCTGTTCTCCTTCACCAAGT-3’ *Fgf10* RW: 5’-GCCATTGTGCTGCCAGTTAA-3’; *Sp-c* FW: 5’- CCTCAAACG CCTTCTCATCG-3’; *Sp-c* RW: 5’- CAGTGGAGCCGATGGAAAAG-3’; *Sp-b* FW:5’-CCAGAGCCAGATTAACCCCA-3’; *Sp-b* RW:5’- AGAAGTCCTGAGTGTGAGGC-3’; *Sox9* FW: 5’-TATCTTCAAGGCGCTGCAAG-3’; *Sox9* RW: 5’-GATCAACTTTGCCAGCTTGC-3’; *Abca3* FW: 5’-GACCCTCCTGTTCTGTGTCA-3’; *Abca3* RW: 5’-AGAAGTACA GGAAGCCACCC-3’.

### Lysate Preparation and Immunoblotting analysis

Total OCT-frozen lung tissues from 3-week-old mice were lysed in RIPA buffer supplemented with fresh protease inhibitors (PMSF 1 mM, NaF 1 mM, NaVO_3_ 1 mM, Na_4_P_2_0_7_ 5 mM, Apoprotein 2 µg/ml, Leupeptin 5 µg/ml). After a 30’ incubation on ice, lysates were centrifuged for 10 min at 12,000 × rpm and protein concentrations were quantified using Bradford Assay Reagent (Thermo Fisher, #1863028). Protein extracts were separated by SDS-PAGE and transferred into a nitrocellulose membrane. The membrane was incubated overnight with the following primary antibodies: rabbit monoclonal EGFR (1:1000, Cell Signaling Technology, #71655), rabbit polyclonal RAB5C (1:1000, Thermo Fisher Scientific, #PA5101828), mouse monoclonal PANRAS (Ab3) (1:1000, Sigma-Aldrich, #OP40) mouse monoclonal α-Smooth Muscle Actin (α-SMA) (1:1000, Sigma-Aldrich, #A5228), mouse monoclonal vimentin (9E7E7) (1:1000, Santa Cruz #66001). As secondary antibodies were used goat anti-mouse (1:10,000 Bethyl, #A90-516P) and anti-rabbit (1:5000, Bethyl, #A120-201P) conjugated to horseradish peroxidase (Bethyl). Protein signals were developed by ECL detection using a ChemiDoc-It Imaging System (UVP, Upland, CA) instrument. We performed the analysis of proteins using precast gels.

Sample sizes for immunoblotting:

- 3 week-old mice: we analyzed 18 WT and 17 *Sri⁻/⁻* mice for EGFR protein expression; 19 WT and 19 *Sri⁻/⁻* mice for PANRAS protein expression; 13 WT and 12 *Sri⁻/⁻* mice for RAB5c protein expression.
- E13.5 embryos: we analyzed 14 WT and 14 *Sri⁻/⁻* mice for EGFR and RAB5C protein expression and 13 WT and 14 *Sri⁻/⁻* mice for PANRAS protein expression.
- 3 month-old mice: we analyzed 13 WT and 12 *Sri⁻/⁻* mice for α-SMA and 5 WT and 6 *Sri⁻/⁻* mice for Vimentin protein expression.
- To quantify the protein expression, we performed densitometric analysis using the stain-free gel technology which allows to the rapid visualization of proteins: polyacrylamide gel contains a proprietary trihalo compound to make proteins fluorescent directly in the gel without traditional staining methods like Coomassie (Biorad). For each gel, the first WT lysate was used as a reference control, and all remaining WT and *Sri⁻/⁻* lysates were normalized to this control.
- Representative Western blot images are shown in the figure.

### Immunofluorescence

For immunofluorescence in mice, lung tissues were covered in OCT mounting medium and frozen in liquid nitrogen precooled isopentane. Ten-micrometer cryosections were fixed in 4% paraformaldehyde for 5’, washed in PSB and permeabilized in 0.1% Triton X-100 in PBS. Then, sections were blocked in 5% goat serum for 1 h and incubated overnight at 4 °C with primary antibody rabbit polyclonal EPCAM/CD326 (1:100 in 1%BSA-PBS solution) (Proteintech, #21050-1-AP), rabbit polyclonal SFTPB (1:100 in 1%BSA-PBS solution) (Thermo Fisher Scientific, #PA542000), rabbit polyclonal E-cadherin (1:100 in 1%BSA-PBS solution) (Proteintech, #20874-1-AP), mouse monoclonal E-cadherin (1:100 in 1%BSA-PBS solution) (BD Bioscience, #610181), mouse monoclonal α-Smooth Muscle Actin (1:100 in 1%BSA-PBS solution) (Sigma-Aldrich, #A5228), rabbit polyclonal EGFR (1:100 in 1%BSA-PBS solution) (Proteintech, #30847-1-AP) rabbit polyclonal RAB5C (1:100 in 1%BSA-PBS solution) (Thermo Fisher Scientific, #PA5101828), mouse monoclonal PANRAS (Ab3) (1:100 in 1%BSA-PBS solution) (Sigma-Aldrich, #OP40) followed by incubation with Alexa fluor 488 (rabbit)-conjugated secondary antibodies (1:500 in 1%BSA-PBS solution) (Thermo Fisher Scientific). For phalloidin immunofluorescence, sections were incubated with Rhodamine phalloidin (Thermo Fisher Scientific) diluted in PBS for 30 min. Then, they were counterstained with Hoechst 33342 (Thermo Fisher Scientific, Waltham, MA, USA), mounted with Vectashield (DBA) and visualized under fluorescence confocal microscopy (Zeiss, Wetzlar, Germany). *n* = 4 mice per condition were analysed.

## Results

### Sorcin deficiency disrupts lung development

Lung development involves two critical epithelial processes: branching morphogenesis and alveolarization, both essential for forming the gas-exchange-competent epithelial cells. To elucidate Sorcin’s role in this process, we first examined the expression and localization of Sorcin (*S*RI) in wild-type (WT) mice lung tissue at 3 weeks and 3 months, by confocal analysis. To distinguish epithelial from mesenchymal tissue we used E-cadherin and α-SMA (alpha-smooth muscle actin) markers, respectively. Sorcin exhibited cytoplasmic localization in both cell types, with a predominant expression in airway epithelial cells (Suppl. Figure 1 A-B), suggesting a significant epithelial role in lung development.

**Fig. 1:**
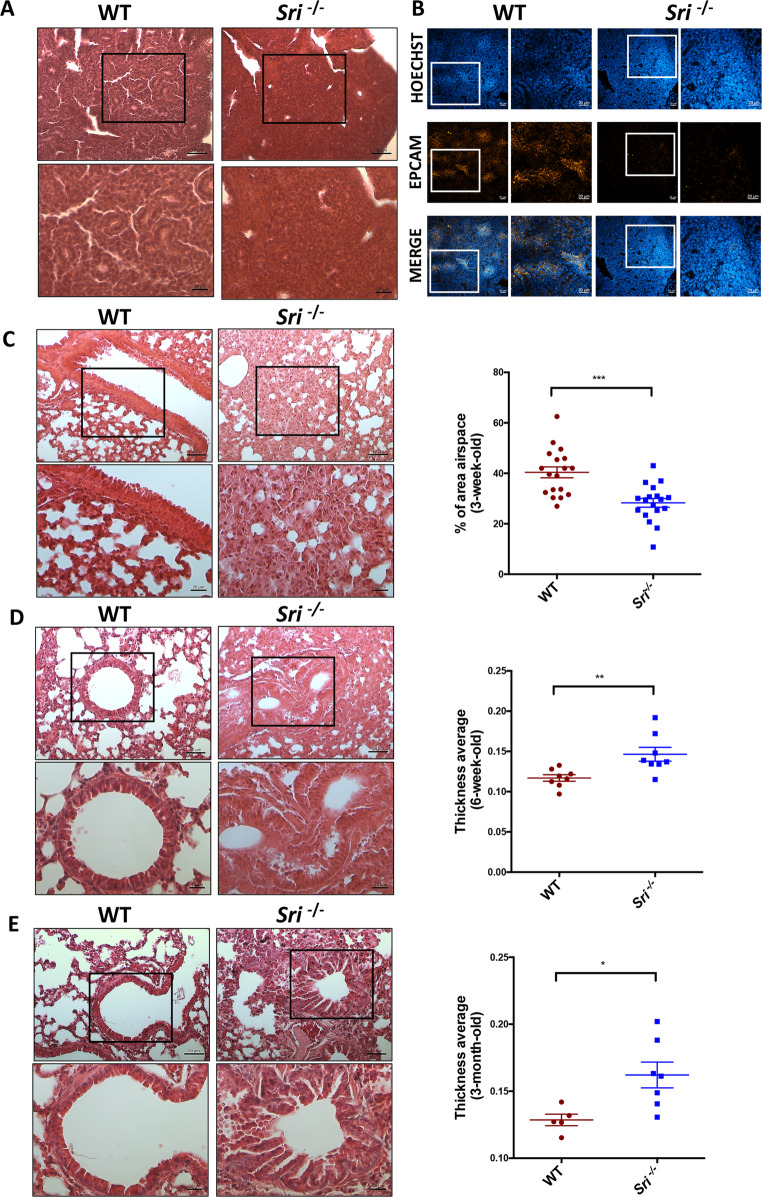
Sorcin deficiency impairs lung morphogenesis and alveolarizationA) Hematoxylin and Eosin (H&E) staining of E16.5 lung sections from wild-type (WT) and Sorcin-deficient (*Sri⁻/⁻ *). *Sri⁻/⁻ *mice exhibited increased cellularity and reduced bronchiole formation compared to WT mice. Scale bars, 20 and 50 μm.B) Confocal immunofluorescence analysis of E16.5 lung sections showing decreased EpCAM (red) expression in *Sri⁻/⁻* mice compared to those WT, indicating a delay in the epithelial development. Scale bars, 20 μm.C) H&E staining of lung sections from 3-week-old WT and *Sri⁻/⁻ *mice. *Sri⁻/⁻ *lungs exhibit impaired bronchiole development, thickened alveolar walls, and reduced alveoli formation compared to WT mice. Scale bars, 20 and 50 μm.D-E) H&E staining of lung sections from 6-week (D) and 3-month-old (E) WT and *Sri⁻/⁻* mice: *Sri⁻/⁻ *mice display persistent alveolarization defects and bronchiolar epithelial hyperplasia. Scale bars, 20 and 50 μm.Quantitative analysis: the ratio of air-filled space to total lung area and average bronchiolar thickness were performed using ImageJ software. Data are presented as mean ± SEM. Statistical significance was assessed by Student’s t-test: *p < 0.05, **p < 0.01, ***p < 0.001. n = 5 mice per group, 10 lung sections per mouse.

We analyzed lung tissue from WT and *Sri*^−*/−*^ mice at various developmental stages: embryonic (E13.5 and E16.5) and adult (3 weeks, 6 weeks, and 3 months). Morphological analysis was performed using hematoxylin and eosin (H&E) staining. While E13.5 embryos showed no significant alterations (data not shown), changes appeared in E16.5 embryos. Lung sections from *Sri*^−*/−*^ mice displayed increased cellular density and reduced tubule formation compared to WT mice, indicating impaired epithelial branching morphogenesis (Fig. 1A). Accordingly, confocal analysis showed a reduced expression of EpCAM protein (Epithelial Cell Adhesion Molecule), a cell surface marker of epithelial progenitor cells, in *Sri*^−*/−*^ compared to WT mice, indicating a developmental delay in airway epithelial morphogenesis (Fig. 1B). These findings are consistent with Sorcin’s predominant expression in the epithelial component of WT mice, as shown in Suppl. Figure 1A-B.

At 3 weeks, *Sri*^−*/−*^ mice maintained a high cellular density and exhibited irregular, thinner alveolar walls with fewer septations compared to WT mice (Fig. 1C). Quantification of air-filled space relative to total lung area further highlighted these differences in alveolarization. Similar defects persisted in 6-week and 3-month-old *Sri*^−*/− −*^ mice. Additionally, *Sri*^−*/−*^ mice displayed hypercellularity around bronchioles (Figs. 1D-E), a finding corroborated by bronchial wall thickness quantification. This hypercellularity may indicate cellular hyperplasia, potentially associated with pathological conditions and inflammation.

### Sorcin regulates genes involved in lung branching morphogenesis and alveolarization

**Fig. 2:**
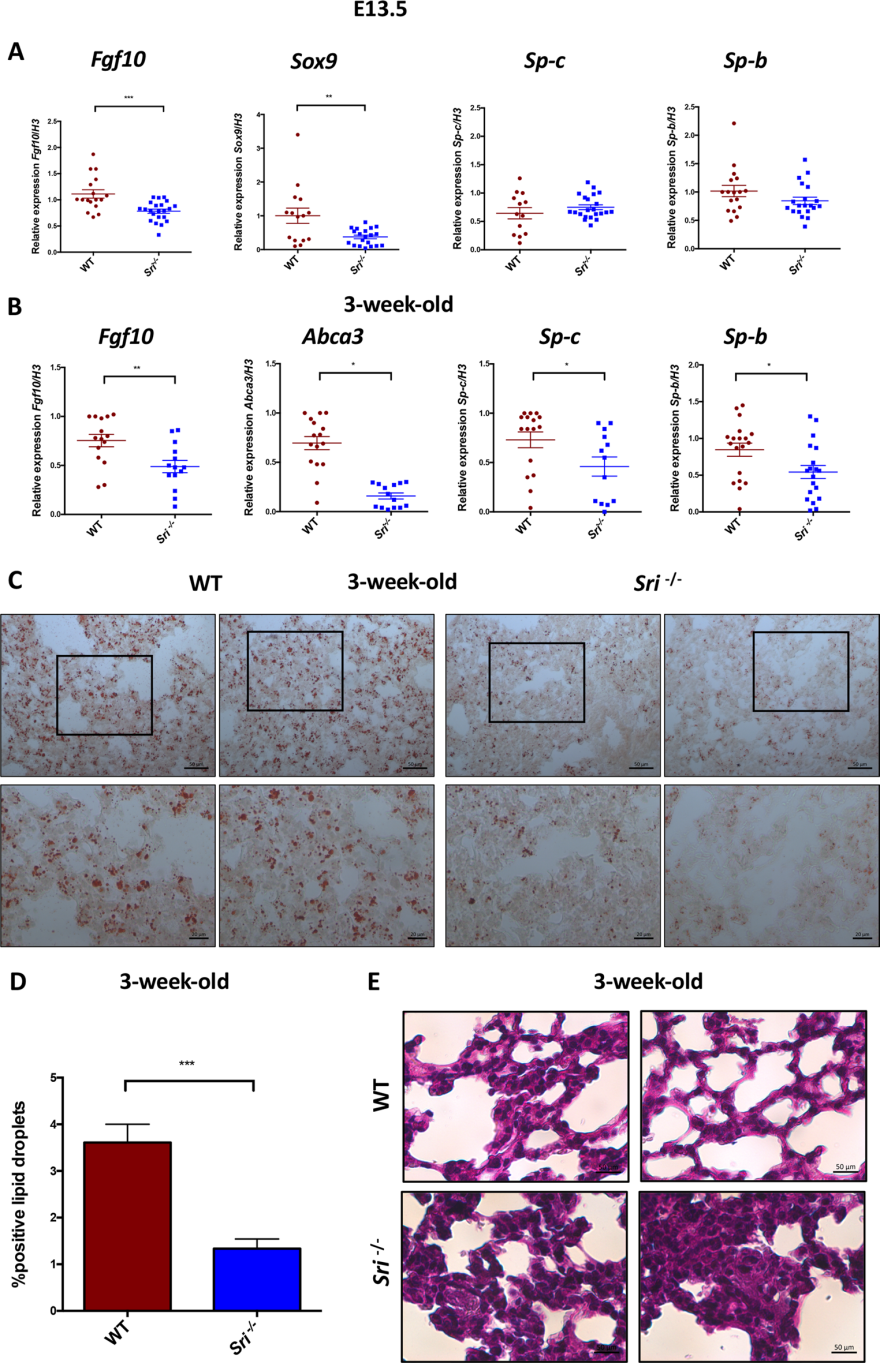
Impaired branching morphogenesis and surfactant production in *Sri*^*−/−*^ mice lungs **A**) E13.5 lung tissue: Embryonic *Sri*^*−/−*^ mice show significantly decreased expression of branching morphogenesis genes (*Fgf10*, *Sox9*), compared to WT mice. Surfactant genes (*Sp-b*, *Sp-c*) exhibit no significant difference between the two groups at this stage **B**) 3-week-old lung tissue: significantly reduced expression of surfactant genes (*Sp-b*, *Sp-c*), and of the phospholipid transporter A*bca3* is observed in *Sri*^*−/−*^ mice compared to WT controls. Data are presented as mean ± SEM. *p < 0.05; **p < 0.01; ***p < 0.001 (determined by Student’s t-test) **C**) Oil Red O staining of lung sections from 3-week-old WT and *Sri*^*−/−*^ mice: *Sri*^*−/−*^ mice exhibit decreased lipid droplet accumulation, indicative of impaired total lipid surfactant content, compared to WT. Scale bars, 20 and 50 μm **D**) Quantification of lipid droplets from Oil Red O stained sections. Data are presented as mean ± SEM. ***p < 0.001 (Student’s t-test) **E**) Periodic acid-Schiff (PAS) staining of lung sections from 3-week-old WT and *Sri*^*−/−*^ mice. *Sri*^*−/−*^ mice show increased glycogen staining, indicative of pneumocyte immaturity, compared to WT. Scale bars, 50 μm

Given Sorcin’s observed role in lung epithelium development, we investigated the underlying molecular mechanisms. Since branching morphogenesis occurs during the pseudoglandular stage of lung development (E10.5-E16.5), we performed gene expression analysis at embryonic day E13.5. RT-qPCR revealed significant downregulation of the critical transcription factors *Fgf10* and *Sox9*, both essential for initial bud formation, in *Sri*^−*/−*^ mice compared to WT. As alveolarization occurs in later stages of lung development, surfactant protein genes S*p-b* and *Sp-c* showed no significant expression differences between the two groups at the embryonic stage (Fig. 2A). However, at 3 weeks, *Sri*^−*/−*^ mice exhibited significant downregulation of *Abca3*, *Sp-b*, and *Sp-c* expression, indicating deficiencies in alveolar surfactant transport and secretion (Fig. 2B). These findings also suggest impaired maturation of lung epithelial ATII pneumocytes, which produce and secrete pulmonary surfactant lipids and proteins.

To further investigate Sorcin’s role in alveolarization, we analyzed lung tissue lipid composition. Oil Red O staining revealed a reduced size and number of lipid droplets in *Sri*^−*/−*^ mice, indicating impaired total lipid surfactant content (Fig. 2C). Quantification of lipid droplet accumulation is presented in Fig. 2D. Nile red staining further confirmed this decrease in intracellular lipids in *Sri*^−*/−*^ compared to WT mice (Suppl. Figure 1 C).

Periodic Acid-Schiff (PAS) staining, used to detect glycogen stores, revealed pneumocyte immaturity and abnormal distribution of glycogen in *Sri*^−*/−*^ sections, which showed intense magenta staining throughout the cytoplasm of the cells, unlike the pattern observed in WT (Fig. 2E). Collectively, these data suggest impaired alveolarization due to decreased surfactant production, potentially leading to respiratory dysfunction.

### Sri^−/−^ mice displayed impaired surfactant production and EGFR signaling

**Fig. 3:**
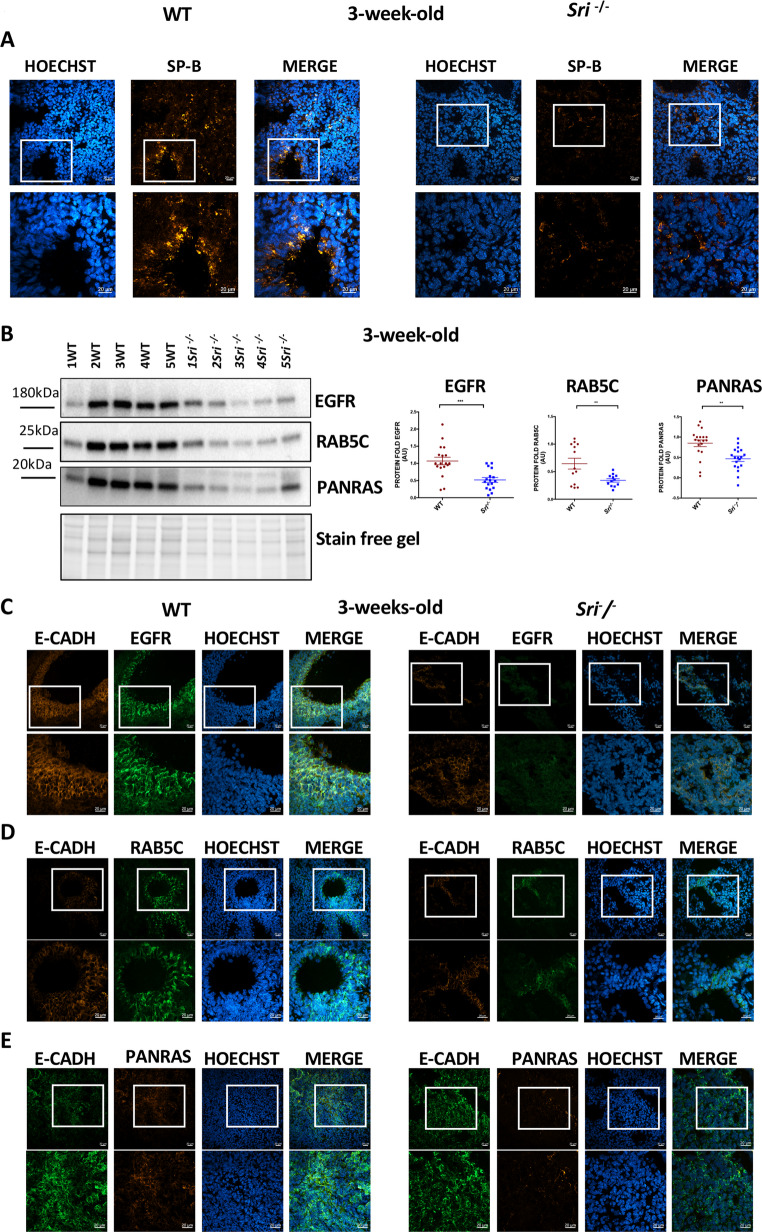
Altered protein expression and EGFR signaling in Sorcin-deficient lung development **A**) Confocal microscopy of 3-week-old lung tissue from WT and *Sri⁻*^*/*^*⁻* mice. *Sri⁻*^*/*^*⁻* lung tissue shows reduced SP-B (red) expression compared to WT. Scale bars, 20 μm **B**) Western blot analysis of lung lysates from 3-week-old WT and *Sri⁻*^*/*^*⁻* mice. *Sri⁻*^*/*^*⁻* mice exhibit decreased EGFR, PANRAS and RAB5C protein levels compared to WT. Densitometry quantification is shown (mean ± SEM). **p < 0.01 (Student’s t-test), ***p < 0.001 (determined by Student’s t-test) **C-E**) Confocal microscopy of 3-week-old lung sections: WT mice show epithelial localization of EGFR (green) (C), RAB5C (green) (D) and PANRAS (red) protein (E). In *Sri⁻*^*/*^*⁻* mice, these proteins are significantly reduced. Scale bars, 20 μm

These molecular and histological findings described above are consistent with those observed in *Egfr*^*−/−*^ mice, which exhibit reduced tubule formation, irregular alveoli with thick walls, undifferentiated epithelial Type II pneumocytes with high glycogen content, and decreased surfactant protein production during alveolarization. Accordingly, confocal microscopy showed reduced SP-B expression in *Sri*^−*/−*^ mice compared to WT at 3 weeks (Fig. 3A), providing insights into Sorcin’s potential role in regulating ATII cell function, surfactant homeostasis and trafficking.

Surfactant secretion involves intracellular processing of SP-B and SP-C proteins from the ER and Golgi to multivesicular bodies (MVBs) and LBs. This trafficking is mediated by early endosomes, which contribute to LB formation and surfactant secretion. To further investigate Sorcin’s role in surfactant secretion and explore the Sorcin (SRI)-EGFR relationship, we analyzed the expression of RAB5C, an isoform of the RAB5 subfamily which play a critical role in regulating early endosome, EGFR and of its downstream effector, PANRAS, both critical for lung development.

Western blot analysis revealed significant reductions of EGFR, PANRAS and RAB5C protein levels in *Sri*^−*/−*^ mice compared to WT mice at 3 weeks (Fig. 3B). Correspondingly, these proteins were also decreased in *Sri*^−*/−*^ mice at the E13.5 embryonic stage (Suppl. Figure 2 A-B).

Considering the epithelial Sorcin expression, we examined the localization of EGFR, RAB5C and PANRAS by confocal analysis, performing a double staining with the epithelial marker E-cadherin (Fig. 3C-E, Suppl. Figure 3 A-C) and the mesenchymal marker α-SMA (Suppl. Figure 3D-E). As a result, we observed a predominant epithelial localization of these proteins in WT mice and an evident reduction in their epithelial expression in *Sri*^−*/−*^ mice (Fig. 3C-E, Suppl. Figure 3 A-C). At 3 weeks, the mesenchymal component remained poorly developed, and low levels of the expression of these proteins were observed both in WT and *Sri*^−*/−*^ mice (Suppl. Figure 3D-E). Interestingly, at 3 months we observed a relevant increase of α-SMA in *Sri*^−*/−*^ mice to WT (Suppl. Figure 4 A-B).

These findings, in addition to the involvement of Sorcin in EGFR regulation, influencing lung development at both physiological level and pathological level [8, 43], suggested a possible impact of Sorcin Knockout mainly on the epithelial development counterpart in the lung tissue.

### Sorcin regulates cytoskeletal airway remodeling and adipose tissue infiltration

Sorcin is highly expressed in vascular smooth muscle, where it modulates Ca²⁺ sparks (decreasing their frequency, amplitude, duration and width) by regulating calcium concentration in the ER, mainly through SERCA activation and RYR inhibition, and therefore modulating intracellular Ca²⁺ levels, which are essential for airway smooth muscle (ASM) contraction [36]. Given the observed increase in bronchiole thickness in.

**Fig. 4:**
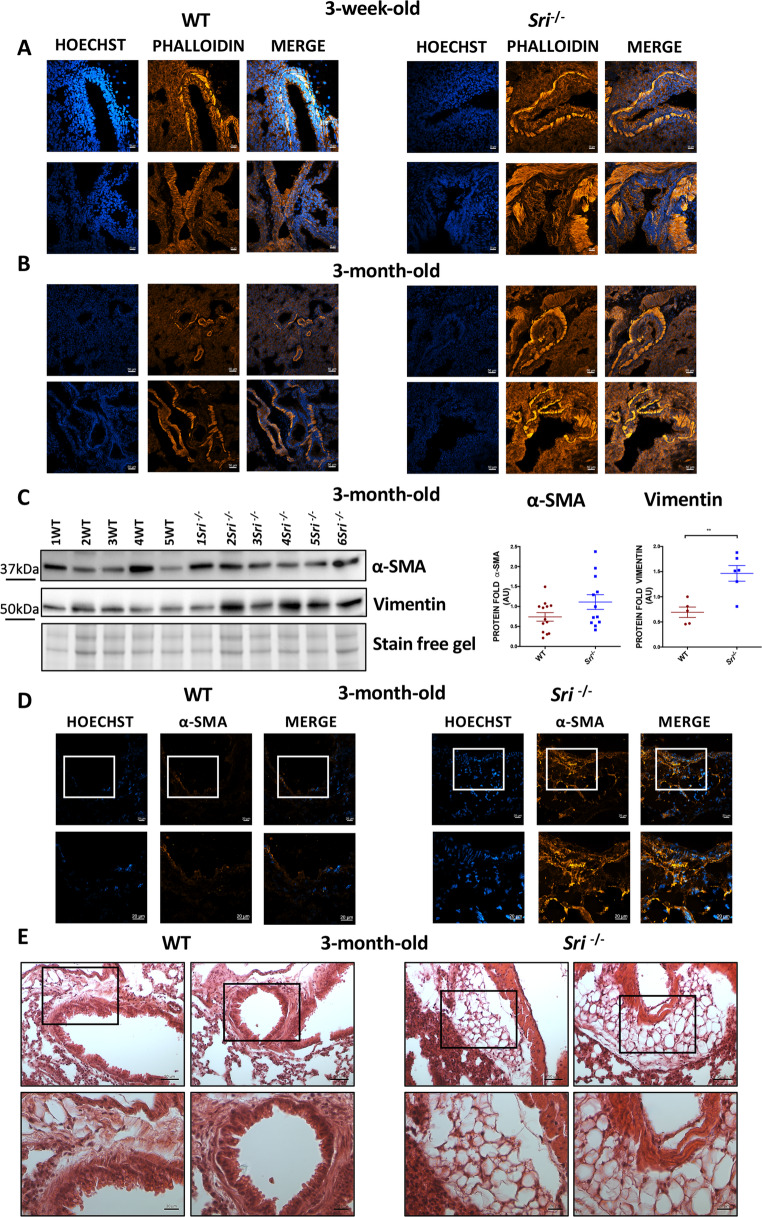
Airway remodeling and adipose tissue accumulation in Sorcin-deficient lungs **A-B**) Confocal microscopy at 3 weeks (A) and 3 months (B) in lung tissue from WT and *Sri⁻*^*/*^*⁻* mice: *Sri⁻*^*/*^*⁻* lung tissue show increased phalloidin expression and thickened airway walls compared to WT. Scale bars, 20 μm **C**) Western Blot analysis of lung lysates from 3-month-old WT and *Sri⁻*^*/*^*⁻* mice. *Sri⁻*^*/*^*⁻* mice exhibit significant increase in vimentin expression and a trend toward higher α-SMA levels in *Sri⁻*^*/*^*⁻* mice compared to WT. Densitometry quantification shown (mean ± SEM). **p < 0.01 (Student’s t-test) **D**) Confocal microscopy of lung sections from 3-month-old WT and *Sri⁻*^*/*^*⁻* mice. *Sri⁻*^*/*^*⁻* mice show an increase of α-SMA (red) compared to WT. Scale bars, 20 μm **E**) H&E staining of lung sections from 3-month-old WT and *Sri⁻*^*/*^*⁻* mice. *Sri⁻*^*/*^*⁻* mice show increased airway adipose tissue accumulation compared to WT. Scale bars, 20 and 50 μm

*Sri*^−*/−*^ mice, we analyzed cytoskeletal remodeling using phalloidin immunofluorescence. Confocal analysis revealed a significant increase in f-actin expression in *Sri*^−*/−*^ mice, suggesting an increased cellular ASM volume compared to WT mice. Notably, these morphological changes were evident as early as three weeks of age and persisted at three months (Fig. 4A-B). Accordingly, we observed a significant increase in vimentin expression and a trend toward higher α-SMA levels in *Sri*^−*/−*^ mice compared to WT. Due to the high variability of α-SMA expression in WT samples, we further highlighted this difference using confocal microscopy, which supports airway smooth muscle (ASM) remodeling and hypercontractility (Fig. 4C-D and Suppl. Figure 5).Taken together, these data suggest that calcium depletion in the ER may affect bronchial contraction, ultimately leading to lung structural and airway tissue remodeling, including an increase of ASM.

Moreover, we observed early and significant fat accumulation *in Sri*^−*/−*^ mice at three months of age, compared to WT (Fig. 4E). Adipose tissue infiltration within the airway epithelium has been reported in obesity-related clinical cases and asthma models, both of which are associated with airway wall thickening and inflammation [45, 46]. These findings suggested that Sorcin deficiency contributes to both cytoskeletal and airway tissue remodeling due to mechanostress events, leading to significant lung structural alterations.

## Discussion

Our previous work demonstrated that Sorcin regulates EGFR signaling in non-small cell lung adenocarcinoma, promoting cellular invasion and migration. We discovered important relationships between calcium homeostasis and EGFR signaling pathways, and identified Sorcin as a key player in EGFR’s physiological and pathological roles, linked to Ca^2+^ dysregulation [43].

Beyond its role in tumorigenesis, EGFR is crucial for lung development, particularly branching morphogenesis and alveolarization. Branching morphogenesis occurs during the pseudoglandular stage (E9.5-16.6 in mice, approximately 16 weeks of gestation in humans), and promotes the formation of a tree-like bronchial system through interactions between epithelial and mesenchymal cells [19, 47, 48]. Alveolarization occurs during the last stages of lung development, specifically during the alveolar stage (P5–P30 in mice, around 20 weeks of gestation in humans, continuing postnatally), involving alveoli formation and maturation, as well as ATI and ATII epithelial cell differentiation. ATII cells play a critical role in producing, secreting, and recycling pulmonary surfactant, which is essential for normal lung function, reducing alveolar surface tension and preventing alveolar collapse.

Histological analysis of embryo and newborn *Egfr*^*−/−*^ mice lungs revealed reduced tubules and increased interstitial mesenchyme during embryonic development, as well as poorly formed, collapsed alveoli characterized by reduced airspace and thickened septa, compared to control mice [8, 9]. In vitro (e.g., in lung explant cultures), EGF treatment enhances branching complexity and number, indicating EGFR’s positive regulatory role in epithelial proliferation and migration [49, 50]. Similarly, our histological analysis of lung tissues from WT and *Sri*^−*/−*^ mice revealed reduced bronchiole formation, increased cellular density and decreased epithelial progenitors - as demonstrated by EpCAM reduction - during the embryonic stage. Consequently, adult *Sri*^−*/−*^ mice displayed abnormal bronchiole maturation with increased of cell layers. This atypical bronchial hyperplasia is a hallmark of inflammation and pulmonary fibrosis, contributing to respiratory distress conditions like asthma and chronic obstructive pulmonary disease (COPD) [51]. Furthermore, persistent hypercellularity and altered alveolar structure with thickened walls indicated impaired alveolarization in *Sri*^−*/−*^ mice. These defects correlate with the predominant epithelial expression of Sorcin in WT mice, indicating that Sorcin’s absence significantly affects alveolarization by impairing the epithelial component.

To characterize Sorcin’s role in lung development, we investigated the molecular pathway and markers involved in branching morphogenesis and alveolarization. The highly coordinated interplay of growth factors and transcription factors, such as *Fgf10* [52–54] and *Sox9* [11], regulates cellular proliferation within tubules, new bud formation, and branching morphogenesis. The surfactant genes *Sp-b* and *Sp-c* encode components of pulmonary surfactant, produced by epithelial ATII cells, which are essential for postnatal alveolar function. Consistently, *Sri*^−*/−*^ mice showed significantly reduced *Fgf10* and *Sox9* levels during embryonic development (E13.5) compared to controls, suggesting impaired branching morphogenesis. As expected, *Sp-b* and *Sp-c* expression was not altered during the embryonic period, given that ATII differentiation occurs later, during the last step of lung development. Since *Fgf10* also drives physiological formation of alveoli [55] and the pool of *Sox9*^+^ progenitors represents precursors of both ATI and ATII cells [56], their reduction suggested alterations in mature lung structures as well. At 3 weeks, *Sri*^−*/−*^ mice exhibited significant decreases in *Sp-b*, *Sp-c*, and *Abca3* (the lipid surfactant transporter in LBs), indicating defective alveolarization, including impaired surfactant trafficking and secretion. These molecular results were consistent with the observed lower lipid surfactant accumulation in *Sri*^−*/−*^ mice, demonstrating impaired secretory pathways and ATII function. Increased glycogen deposits within ATII cells indicated cell immaturity or damage.

ATII cells play a critical role in producing and secreting pulmonary surfactant via LB fusion. The surfactant secretion pathway in ATII cells involves the translocation of the precursor proteins pro-SP-B and pro-SP-C from the ER to the Golgi apparatus, followed by their trafficking to small vesicles and MVBs. Within these compartments, SP-B and SP-C undergo maturation before fusing with LBs. The early endosome pathway, regulated by RAB proteins (including the RAB5 family isoforms RAB5a, RAB5b, and RAB5c), sorts these precursors, directing them to the appropriate intracellular compartments for further processing, including small vesicles and MVBs. Huang et al. showed that a negative variant of RAB5c led to altered early endosome that failed to fuse with proSP-B- or proSP-C-containing nascent sorting vesicles, impairing surfactant protein processing and trafficking, and causing interstitial lung disease [57]. Consistent with this, we observed reduced SP-B and RAB5c protein levels in 3-week-old *Sri*^−*/−*^ mice. Disruption in early endosomal trafficking can impair proper SP-B delivery and processing, potentially leading to surfactant deficiencies and respiratory dysfunction, highlighting the critical role of Sorcin in surfactant secretion.

As noted, impaired EGFR signaling is associated with branching morphogenesis and alveolarization defects. Accordingly, both E13.5 and 3-week-old *Sri*^−*/−*^ mice showed decreased EGFR and impairment of its downstream pathway, including PANRAS protein, compared to WT. These data reinforce the relationship between Sorcin and the EGFR signaling pathway previously demonstrated in our lung adenocarcinoma in vitro study [43]. Moreover, the epithelial localization of EGFR, RAB5C and PANRAS in WT mice - which correlates with epithelial Sorcin expression - and their reduction in the respective component in *Sri*^−*/−*^ mice, suggest that the absence of Sorcin impacts on the expression of these proteins, affecting the epithelial lung development. These data are also consistent with the decreased EpCAM expression observed in embryonic *Sri*^−*/−*^ mice, as well as with the reduced surfactant production by epithelial ATII cells. At three months, *Sri*^−*/−*^ mice display an increase in mesenchymal tissue compared to WT mice. Accordingly, calcium oscillations between ER and cytosol are essential for muscle contraction [58]. In airway smooth muscle (ASM), force generation begins with the myosin light chain kinase (MLCK) phosphorylation and spreads via actin filament polymerization. Alterations in the contractile apparatus and mechanotransduction pathways can lead to asthma, a chronic inflammatory disease of the airways [59]. Increased cytosolic Ca²⁺ activates MLCK, leading to ASM contraction and airway narrowing [60]. Many experimental asthma mouse models show airway remodeling and hyperresponsiveness, including increased ASM contractility due to actin cytoskeletal remodeling and cell proliferation [61]. Mahan et al. demonstrated that SERCA2 downregulation leads to airway remodeling, highlighting the importance of calcium homeostasis - including the calcium release from ER- for normal lung function. ASM of asthmatics patients showed reduced SERCA2 expression [62]. Accordingly, 3-week and 3-month-old *Sri*^−*/−*^ *⁻* mice showed increased f-actin filaments and α-smooth muscle actin expression around the peribronchial region, suggesting cytoskeletal airway remodeling and excessive bronchial contraction, likely due to altered intracellular calcium homeostasis.

Asthma symptoms worsen in obese patients, which often exhibit increased airway-associated adipose tissue [46, 63]. This fat accumulation may contribute to increased bronchial wall thickness and inflammation [45], potentially explaining the high adipose tissue infiltration observed during lung development in our *Sri*^−*/−*^ mice.

## Conclusions

This study provides novel insights into Sorcin’s role in fetal lung development, demonstrating its impact on branching morphogenesis and alveolarization, including surfactant production and secretion, characterized by impairment of epithelial tissue. Additionally, our findings suggest that altered intracellular calcium levels may directly affect EGFR signaling and bronchial contractility, leading to airway cytoskeletal remodeling and adipose tissue accumulation, which can exacerbate airway obstruction. These combined changes may contribute to the development of respiratory disorders.

This study also reveals novel key cell signaling mechanisms regulating fetal lung development, providing a basis for innovative strategies to enhance lung maturation in clinical conditions where development is compromised. Additionally, it addresses critical challenges by identifying approaches to prevent surfactant-related disorders, such as respiratory distress syndrome, ultimately contributing to improved therapeutic outcomes.

## Supplementary Information

Below is the link to the electronic supplementary material.

Supplementary figure 1 (PNG 2.31 mb)

High Resolution Image (TIF 27.9 mb)

Supplementary figure 2 (PNG 356 kb)

High Resolution Image (TIF 23.1 mb)

Supplementary figure 3 (PNG 2.75 mb)

High Resolution Image (TIF 29.4 mb)

Supplementary figure 4 (PNG 143 kb)

High Resolution Image (TIF 27.6 mb)

Supplementary figure 5 (PNG 2.21 mb)

High Resolution Image (TIF 21.9 mb)

## Data Availability

The data that support the findings of this study are available from the corresponding author upon reason- able request.

## Abbreviations

ABCA3: ATP-binding cassette sub-family A member 3
α-SMA: alpha-smooth muscle actin
ASM: airway smooth muscle
ATI: alveolar type I
ATII: alveolar type II
COPD: chronic obstructive pulmonary disease
EpCAM: Epithelial Cell Adhesion Molecule
ER: Endoplasmic Reticulum
EGFR: Epidermal growth factor receptor
FFPE: Formalin-fixed paraffin-embedded
KO: Knockout
LBs: Lamellar bodies
LVCC: L-type voltage-gated calcium channel
MLCK: Myosin light chain kinase
MVBs: Multivesicular bodies
NCX: Na+-Ca2 + exchanger
NSCLC: Non-small-cell lung carcinoma
PAS: Periodic acid-Schiff
PMCA: Plasma membrane Ca2 + pump
RDS: Respiratory distress syndrome
RYRs: Ryanodine receptors
SERCA: Sarco/endoplasmic reticulum Ca2 + pump
SP-B: Surfactant protein B
SP-C: Surfactant protein C
SRI: Sorcin
TTF-1: Thyroid transcription factor-1
