# Supplementary figures and images for: Sorcin regulates alveolarization and airway tissue remodeling during lung morphogenesis
Source: Cell Mol Life Sci. 2025 Oct 28;82(1):367. doi: 10.1007/s00018-025-05870-y (PMC12569328; doi:10.1007/s00018-025-05870-y)

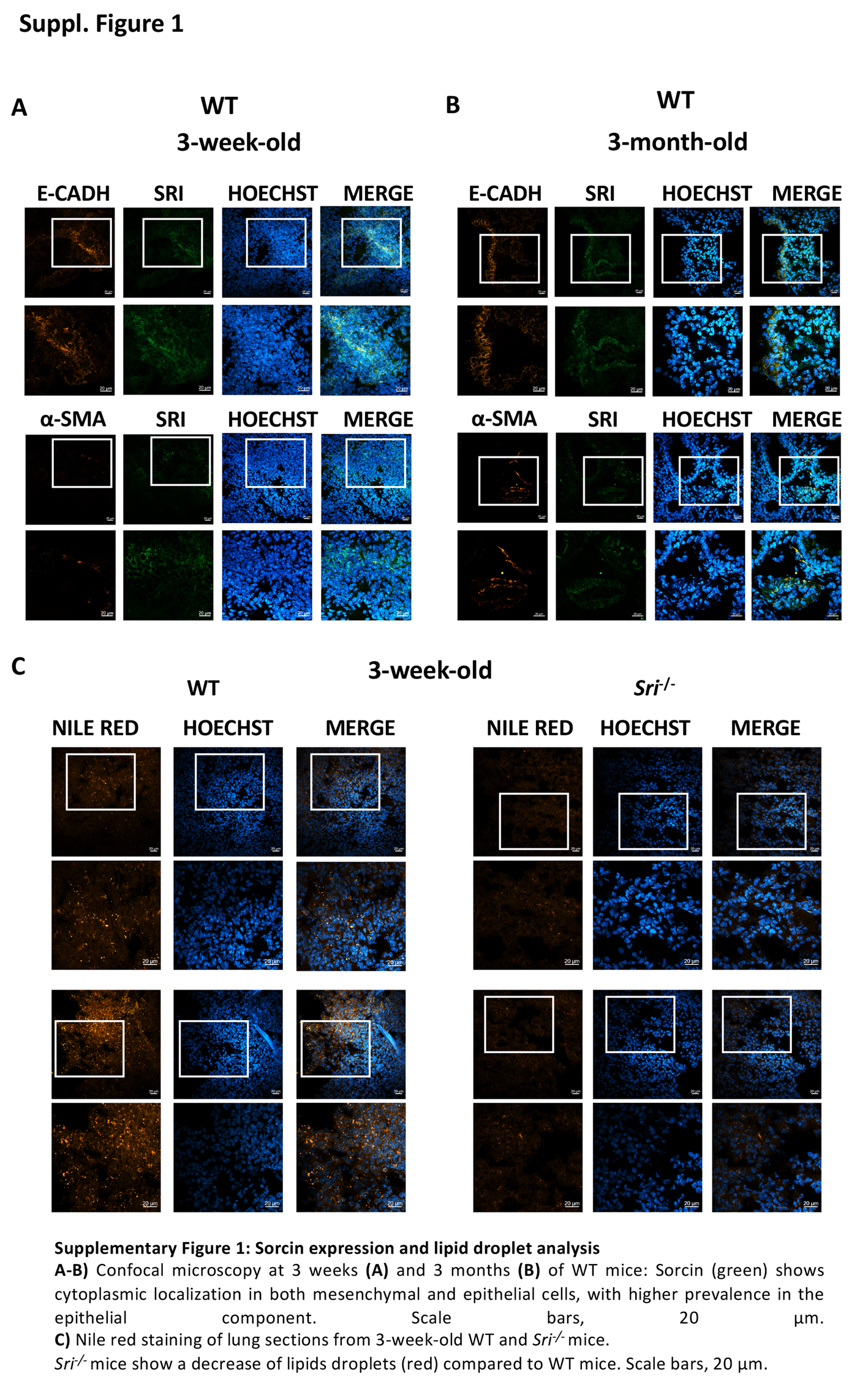

Supplement: Supplementary file 1 — (PNG 2.31 mb) [file 18_2025_5870_Fig5_ESM.png]

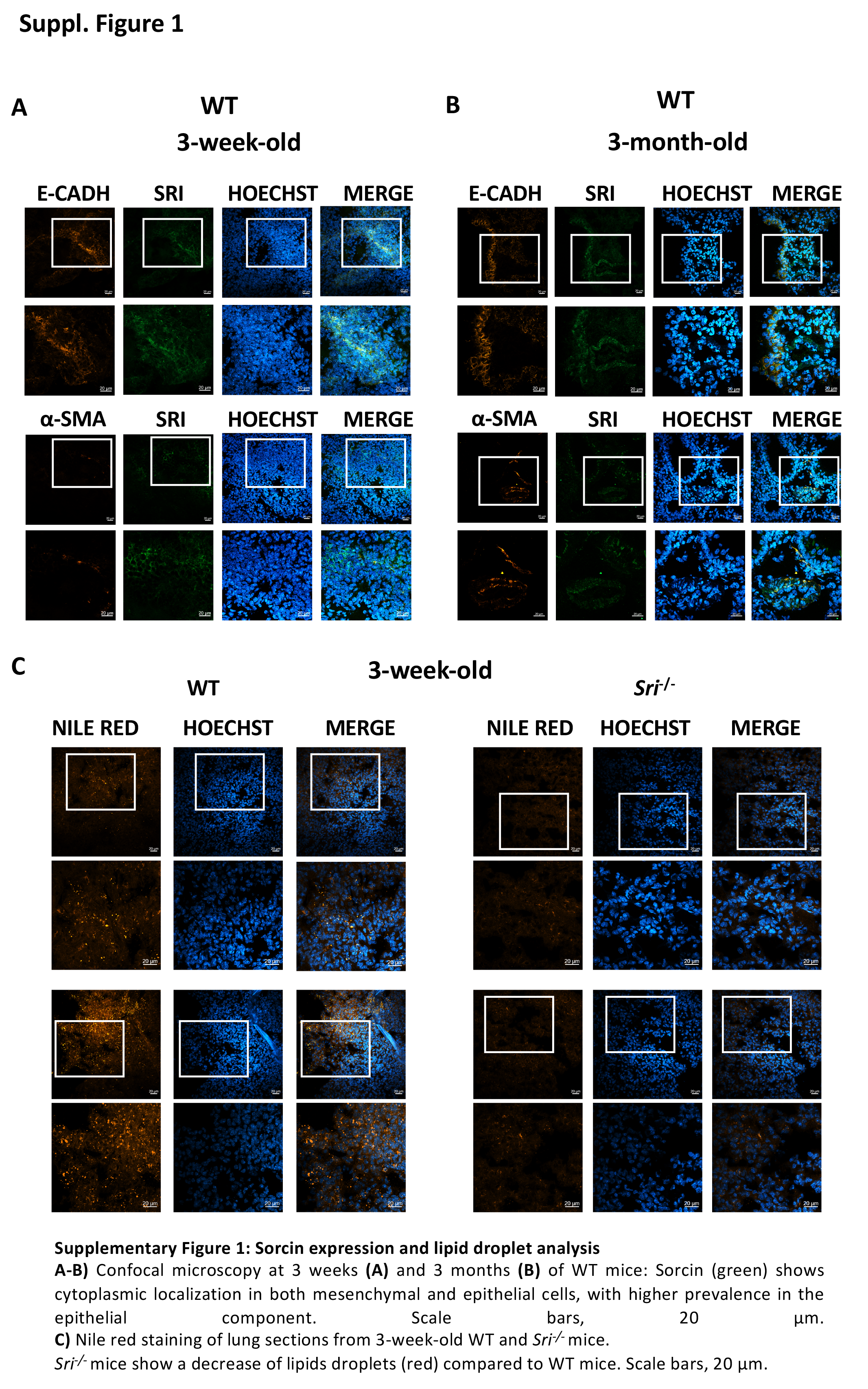

Supplement: Supplementary file 2 — High Resolution Image (TIF 27.9 mb) [file 18_2025_5870_MOESM1_ESM.tif]

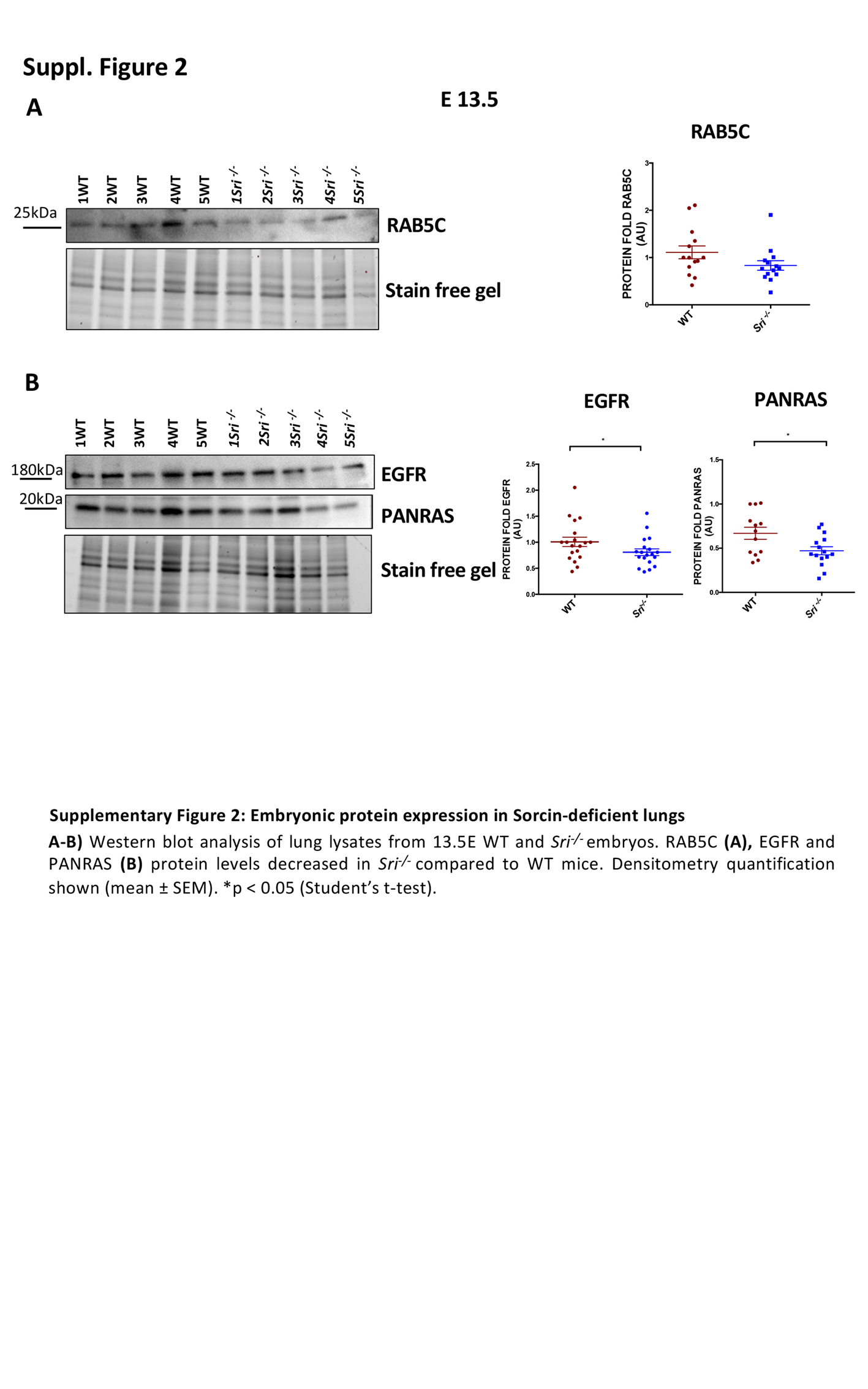

Supplement: Supplementary file 3 — (PNG 356 kb) [file 18_2025_5870_Fig6_ESM.png]

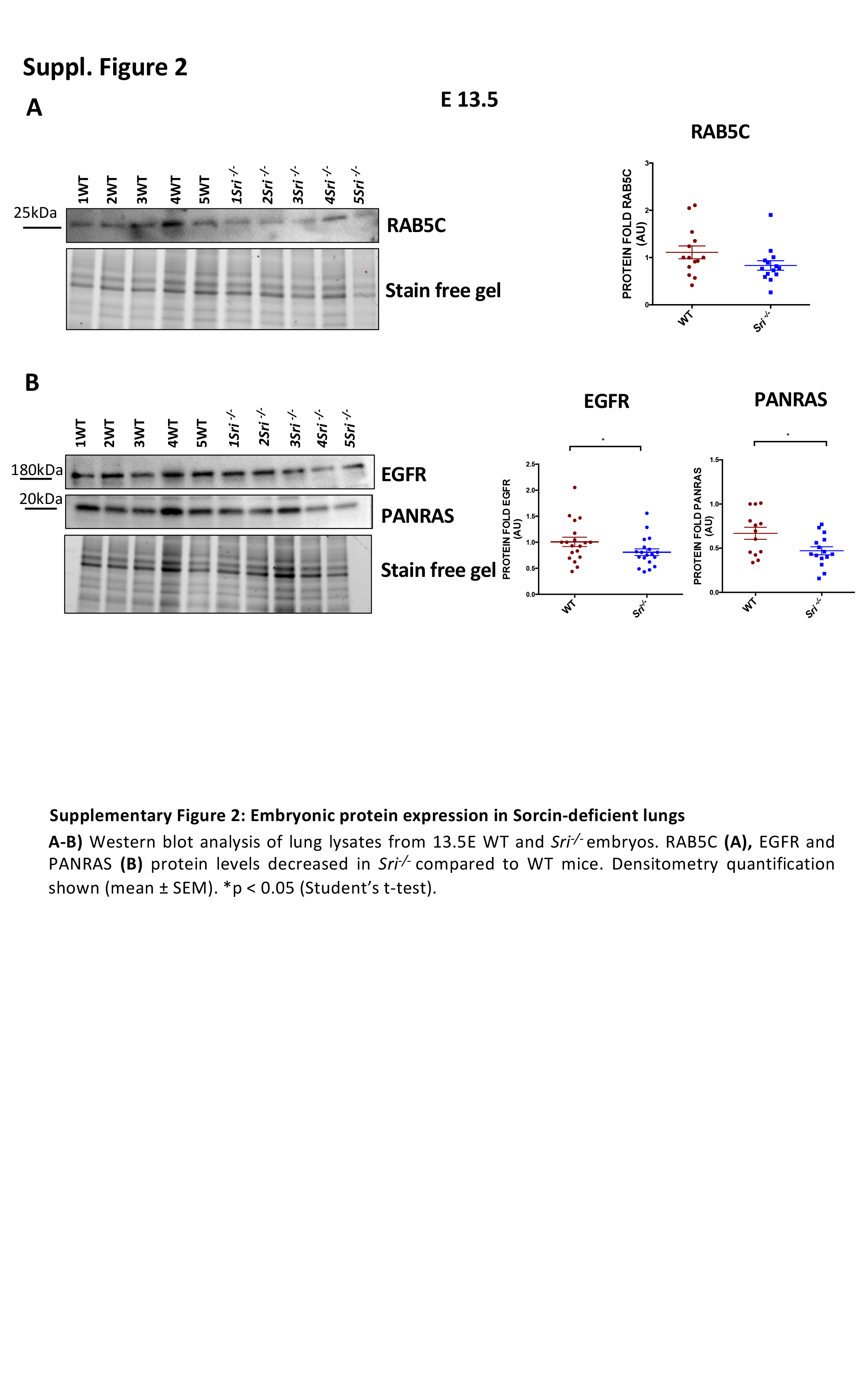

Supplement: Supplementary file 4 — High Resolution Image (TIF 23.1 mb) [file 18_2025_5870_MOESM2_ESM.tif]

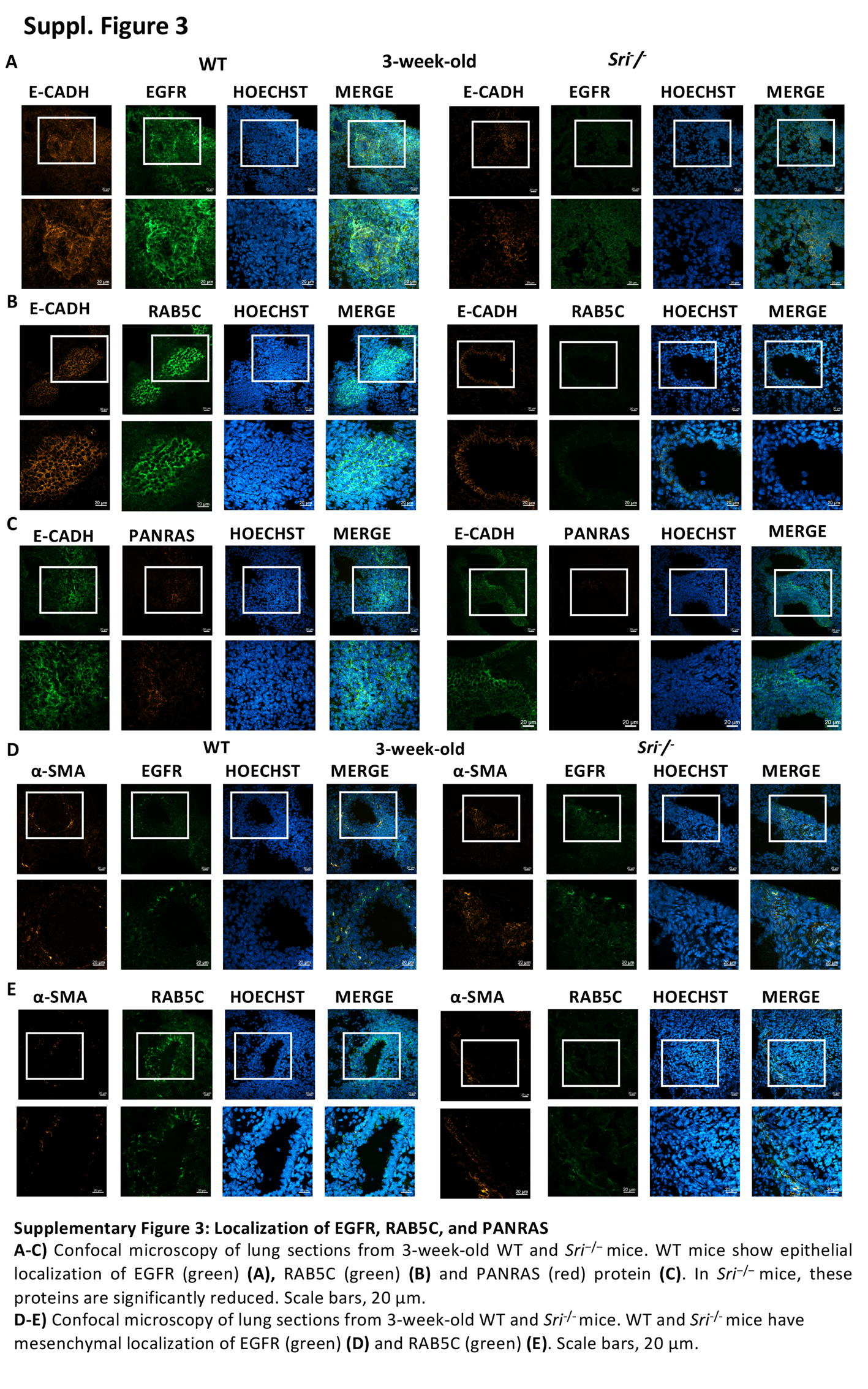

Supplement: Supplementary file 5 — (PNG 2.75 mb) [file 18_2025_5870_Fig7_ESM.png]

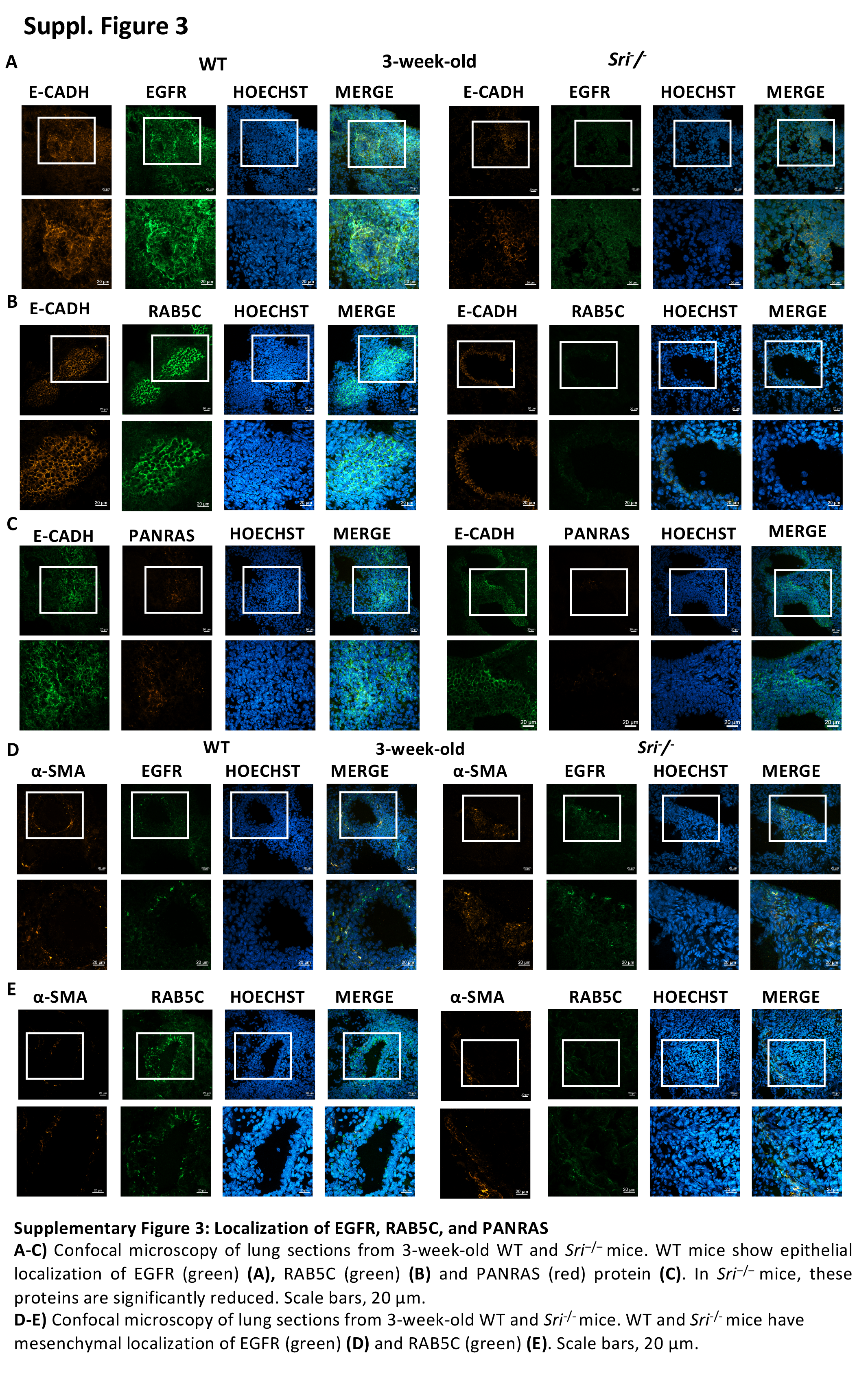

Supplement: Supplementary file 6 — High Resolution Image (TIF 29.4 mb) [file 18_2025_5870_MOESM3_ESM.tif]

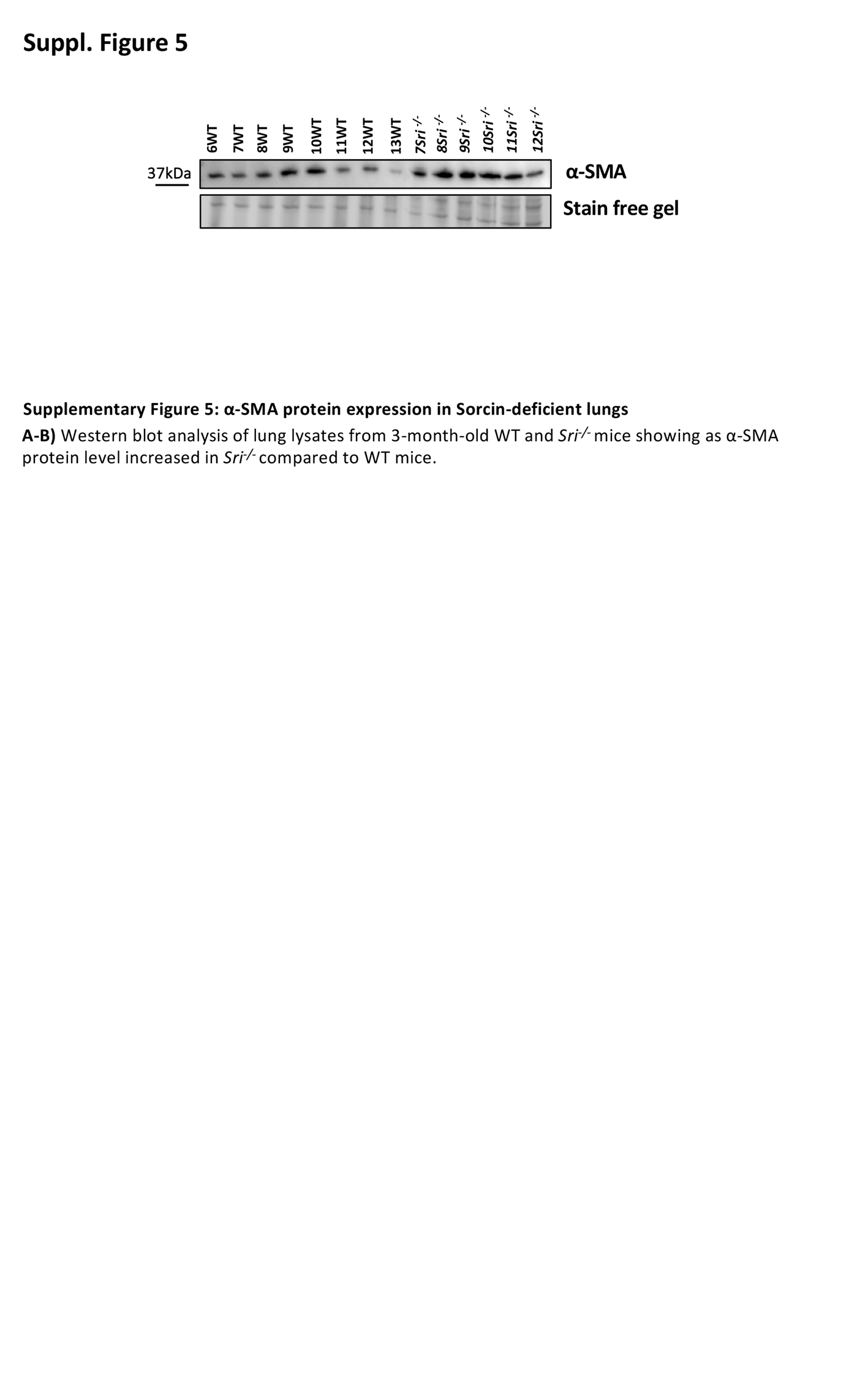

Supplement: Supplementary file 7 — (PNG 143 kb) [file 18_2025_5870_Fig8_ESM.png]

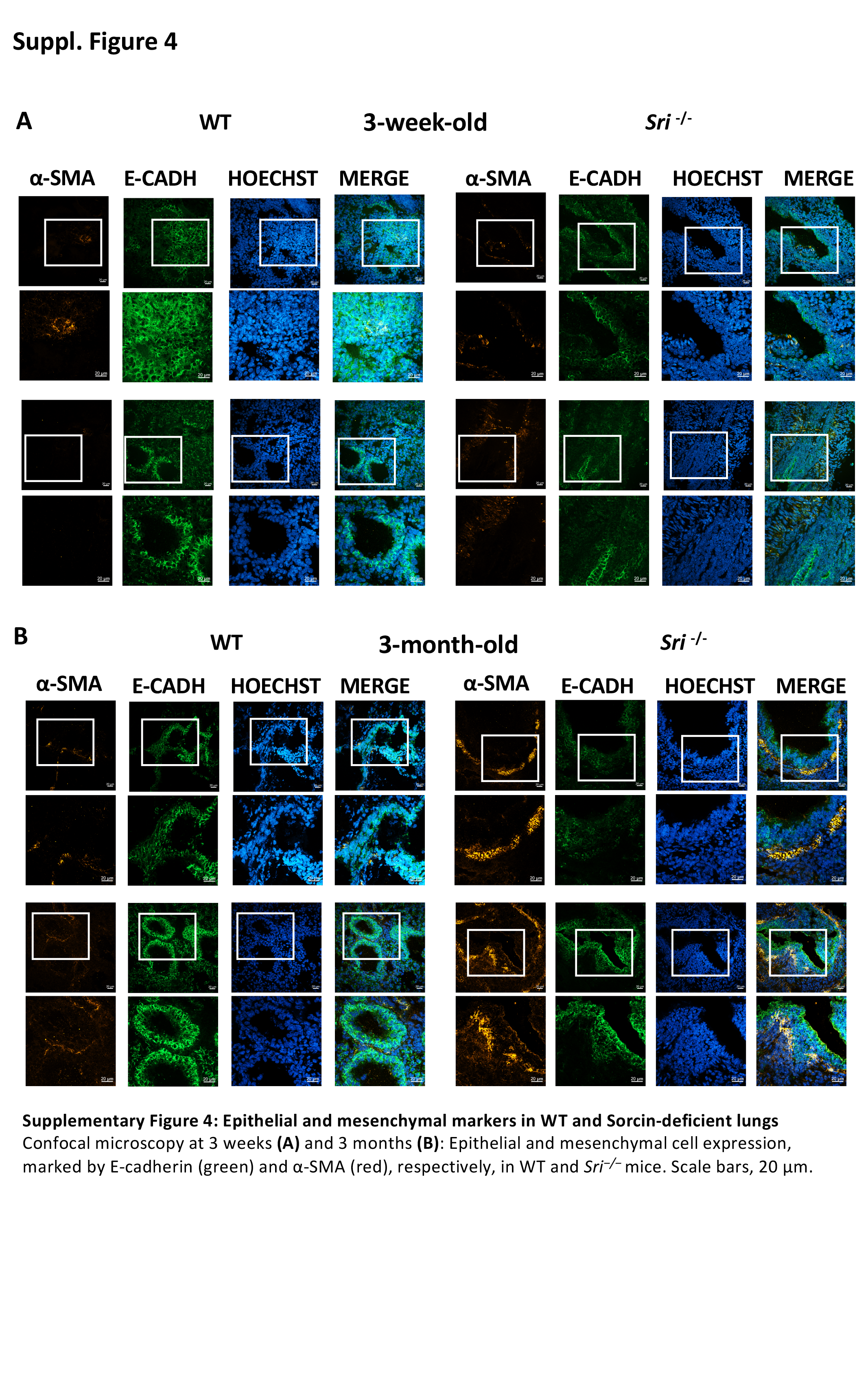

Supplement: Supplementary file 8 — High Resolution Image (TIF 27.6 mb) [file 18_2025_5870_MOESM4_ESM.tif]

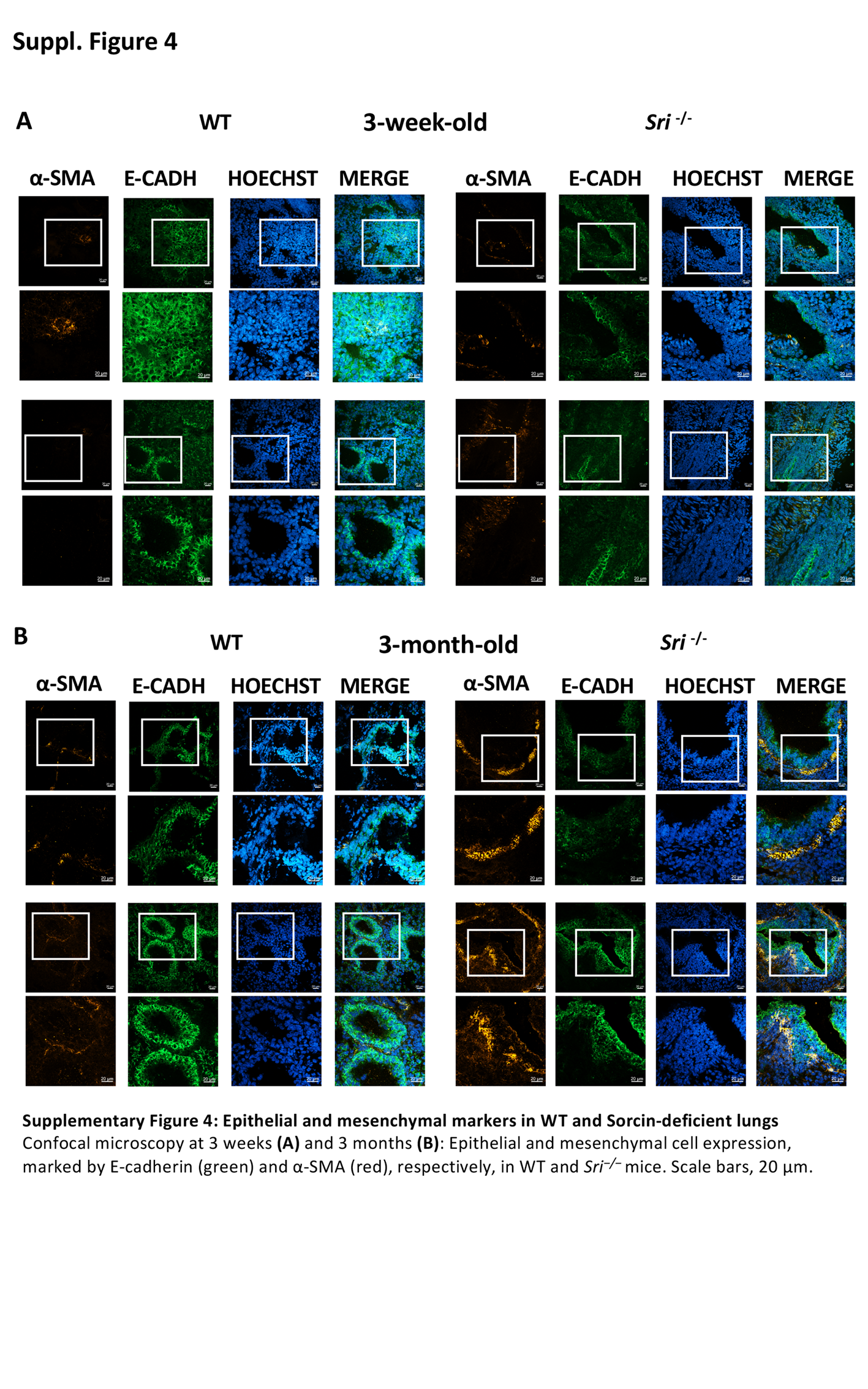

Supplement: Supplementary file 9 — (PNG 2.21 mb) [file 18_2025_5870_Fig9_ESM.png]

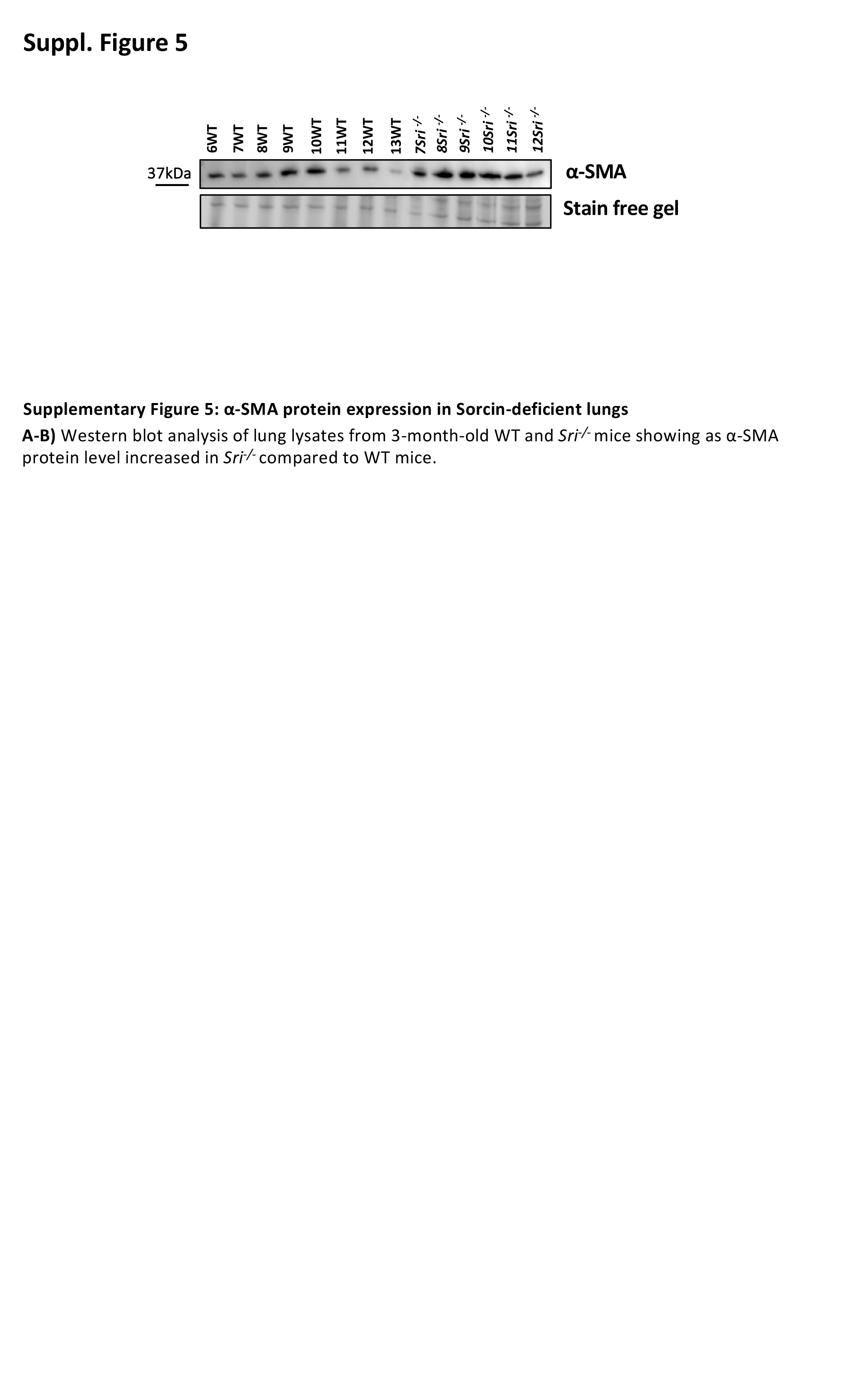

Supplement: Supplementary file 10 — High Resolution Image (TIF 21.9 mb) [file 18_2025_5870_MOESM5_ESM.tif]
